# Emerging targeted and cellular therapies in the treatment of advanced and metastatic synovial sarcoma

**DOI:** 10.3389/fonc.2023.1123464

**Published:** 2023-01-25

**Authors:** Joseph R. Fuchs, Brian C. Schulte, Jeffrey W. Fuchs, Mark Agulnik

**Affiliations:** ^1^ Department of Medicine, McGaw Medical Center of Northwestern University, Chicago, IL, United States; ^2^ Department of Medicine, University of California, San Francisco, San Francisco, CA, United States; ^3^ Medical Oncology and Therapeutics Research, City of Hope Comprehensive Cancer Center, Duarte, CA, United States

**Keywords:** synovial sarcoma, soft tissue sarcoma, therapeutics, clinical trial, adoptive cell transfer

## Abstract

Synovial sarcoma is a soft tissue sarcoma accounting for approximately 1,000 cases per year in the United States. Currently, standard treatment of advanced and metastatic synovial sarcoma is anthracycline-based chemotherapy. While advanced synovial sarcoma is more responsive to chemotherapy compared to other soft tissue sarcomas, survival rates are poor, with a median survival time of less than 18 months. Enhanced understanding of tumor antigen expression and molecular mechanisms behind synovial sarcoma provide potential targets for treatment. Adoptive Cell Transfer using engineered T-cell receptors is in clinical trials for treatment of synovial sarcoma, specifically targeting New York esophageal squamous cell carcinoma-1 (NY-ESO-1), preferentially expressed antigen in melanoma (PRAME), and melanoma antigen-A4 (MAGE-A4). In this review, we explore the opportunities and challenges of these treatments. We also describe artificial adjuvant vector cells (aAVCs) and BRD9 inhibitors, two additional potential targets for treatment of advanced synovial sarcoma. This review demonstrates the progress that has been made in treatment of synovial sarcoma and highlights the future study and qualification needed to implement these technologies as standard of care.

## 1 Introduction

Synovial sarcoma (SYN) is a soft tissue sarcoma accounting for 5-14% of all soft tissue sarcomas (1, 2). The incidence of SYN in the United States is approximately 1.42 per million for adults and 0.81 per million for children and adolescents, accounting for roughly 1,000 cases per year (3). SYN presents at an average age of 35-40 years and there is equal distribution of cases between females and males (3–6). SYN most often arises in deep tissues of the extremities but can also present as head and neck, trunk, and lung lesions (3, 7). Epidemiologic studies have found that most patients are diagnosed with local disease while 10-13% of patients initially present with metastatic disease (3, 7).

The diagnosis and staging of SYN involve pathologic and radiographic review. SYN is defined by the presence of translocation of t(X:18) (p11.2;q11.2) using FISH or RT-PCR and is found in more than 95% of tumors (8). This translocation leads to the fusion of genes *SYT* on Chromosome 18 and *SSX* on Chromosome X, which causes production of SS18-SSX1, SS18-SSX2, or SS18-SSX4 (9–11). These oncogenic fusion proteins impact cellular transcription and metabolism, leading to sarcomagenesis.

For localized cases of SYN, initial therapy is most commonly surgical resection with or without radiation therapy. Neoadjuvant or adjuvant chemotherapy is considered in select cases (12, 13). SYN has high metastatic potential with a historic five year metastasis-free survival rate of 50-60% (14). For locally advanced or metastatic disease, first line therapy usually incorporates anthracycline-based chemotherapy with or without ifosfamide (13, 15, 16). SYNs are relatively chemosensitive tumors compared to other soft tissue sarcomas. In primary soft tissue sarcomas, early localized and metastatic recurrence have been found to occur at a median of 38.3 and 41.3 months, respectively (17). In contrast, SYN has been found to have local recurrence at a mean of 43 month and metastatic recurrence at 68 months (18). A review of 15 clinical trials of first-line chemotherapy for SYN has shown a 27.8% response rate compared to 18.8% in other soft tissue sarcomas (19). When comparing SYN to other soft tissue sarcomas, progression free survival (PFS) was 6.3 months versus 3.7 months and overall survival (OS) was 15.0 months versus 11.7 months, respectively (19). Despite this response, however, for those with metastatic disease one year survival remains 59.5% and the median overall survival is 17.0 months (95% CI 14.5-19.5) (6).

Currently, after anthracycline-based chemotherapy, the only other systemic therapy for treatment of advanced or metastatic SYN approved by the FDA is pazopanib. This approval was granted after pazopanib was shown to improve PFS compared to placebo in a population of patients with varying non-adipocytic metastatic soft tissue sarcomas which included 30 patients with SYN (20).

Improved understanding of cellular and molecular processes behind the development of SYN and advancements in knowledge of SYN’s antigen expression will allow for potential targets for treatment of advanced SYN. In this review, we explore emerging therapies in the treatment of advanced and metastatic SYN.

## 2 SYN antigen expression and adopted cell transfer

SYN has been found to express cancer testis antigens (CTAs) (21). CTAs are antigens with predominant expression in the testis and are not normally found in somatic tissue (22). CTAs are a potential target for treatment of malignancies as they elicit humoral and cellular immune responses (23). SYN has been found to have high expression of CTAs (24). Due to their high expressivity, selectivity, and immunologic response, CTAs have been identified as potential targets for treatment of SYN.

**Figure 1:**
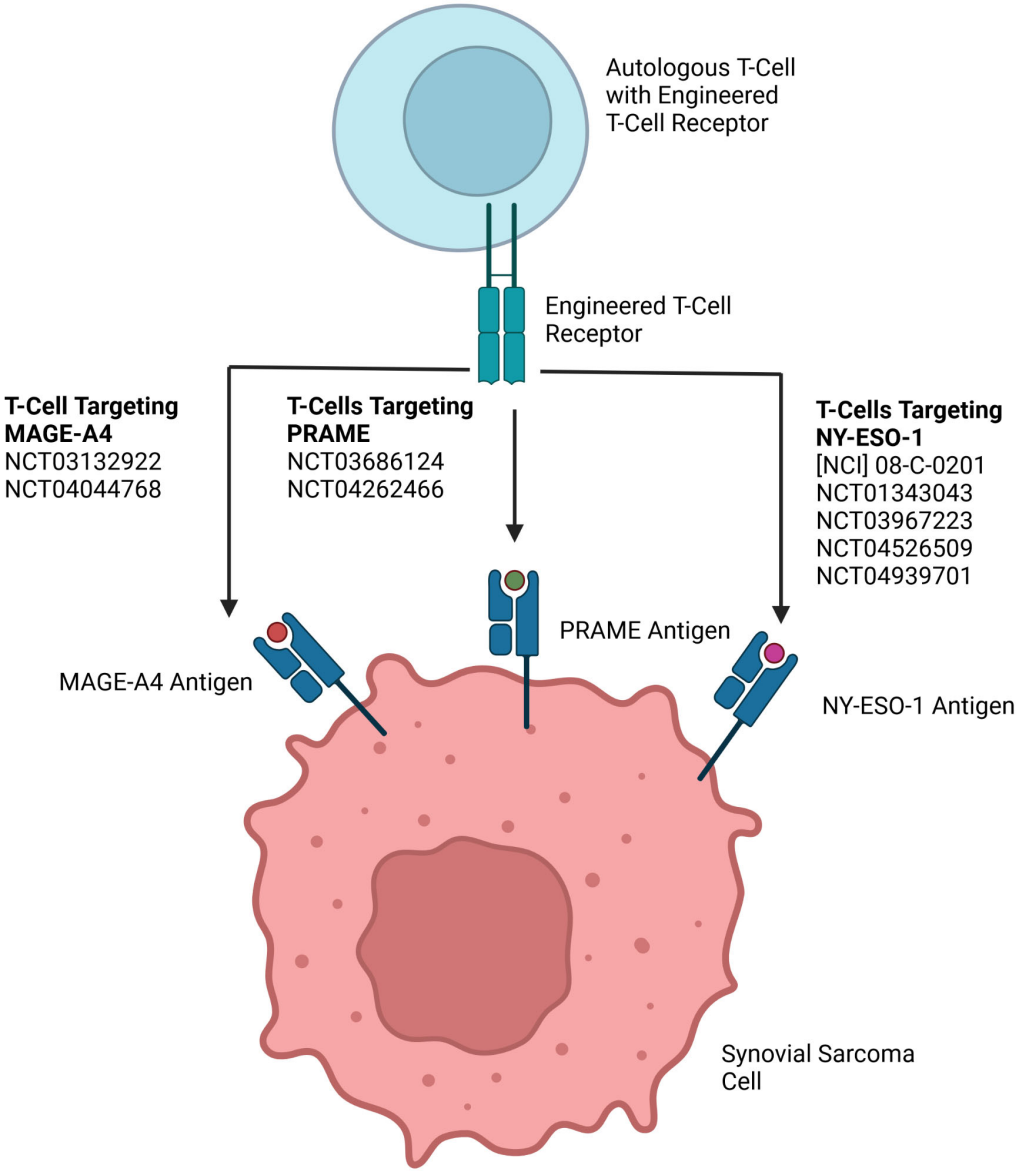
Adopted Cell Transfer engineered T-cell receptors targeting CTAs for treatment of SYN.

Adopted Cell Transfer (or Therapy) (ACT) uses tumor antigen specific T-cells obtained from resected tumor specimens which are expanded *in vitro* and then infused for treatment of cancers (25, 26). One challenge of this treatment is that not all resected tumors allow for the expansion of autologous tumor infiltrating T-cells (27). This obstacle, along with variable quantities of T-cells within tumors, has prompted study of genetically engineered T-cell receptors to target cancer specific antigens. These T-cells are obtained through the harvesting of patient autologous T-cells which are then genetically modified to express a T-cell receptor for a cancer antigen. This technology is currently in development for the treatment of SYN targeting CTAs (**Figure 1**).

### 2.1 NY-ESO-1 adopted cell transfer

New York esophageal squamous cell carcinoma-1 (NY-ESO-1) is a CTA that was first described from serological analysis of recombinant cDNA expression libraries (SEREX) of esophageal squamous cell carcinoma (28). NY-ESO-1 is expressed in approximately 80% of SYNs. The immunogenicity of NY-ESO-1 has led to its consideration as a target for treatment of SYN (23, 29).

In 2011, Robbins et al. conduced the first clinical trial of autologous T-cells genetically engineered to have a specific T-cell receptor for NY-ESO-1 for patients with metastatic melanoma and SYN (**Table 1**) (38). The trial utilized a retroviral vector to create CD4+ and CD8+ autologous T-cells with a T-cell receptor that recognized the SLLMWITQC peptide of NY-ESO-1 for HLA-A*0201, named IG4- α95:LY ([NCI] 08-C-0121) (30). These T-cells were expanded *in vitro* and then transferred to patients after nonmyeloablative chemotherapy along with IL-2. This trial demonstrated objective clinical response in four out of six patients with SYN. The study was then expanded for 12 additional patients with SYN (31). Results demonstrated complete response for one patient and partial response observed in 10 of the 18 total patients with SYN. The three-year survival rate was 38% and the five-year survival rate was 14%.

**Table 1:** Clinical trials completed or with preliminary results for treatment of SYN.

Target	Treatment, Population	Trial, Publication year	Phase, Study Size	Summary
NY-ESO-1	Anti-NY-ESO-1 T-cells, HLA- A*0201, Metastatic SYN or melanoma	[NCI] 08-C-0201, 2015 (30, 31)	II, 18 with SYN	1 complete response, 10 partial responses. Three-year survival 38%, five-year survival 14% AEs: 100% of patients with neutropenia and thrombocytopenia
	Autologous NY-ESO-1^c259^ T-cells, HLA- A*02, Unresectable, metastatic, or recurrent SYN	NCT01343043, 2020 (26, 32)	I, 45	1 complete response (34 weeks), 14 partial responses (across four cohorts) AEs: 40% with Grade 3 or higher hematologic AEs, 44% with cytokine release syndrome (4 patients Grade 3 or higher)
PRAME	Anti-PRAME T-cells, HLA matching, PRAME expression solid tumors	NCT03686124*, 2021 (33)	I, 12	6 patients with partial response, 6 with stable disease (3 patients with SYN) AEs: cytopenia, cytokine release syndrome and neurotoxicity (Grade 1-2), one dose limiting toxicity
MAGE-A4	Autologous MAGE-A4^c1032^ T-Cells, HLA-A*02, expression of MAGE-A4	NCT03132922, 2020 (34, 35)	I, 28	7 patients with partial response, 11 with stable disease AEs: No dose limiting toxicities, >30% Grade 3 or higher hematologic AEs, two trial related deaths due to aplastic anemia and cerebral vascular accident
	Anti-MAGE-A4 T-cells, HLA-A*02, Advanced SYN or myxoid/round cell liposarcoma	NCT04044768*, 2022 (36, 37)	II, 51	36.2% response rate, median duration or response 52 weeks (8.29 – 75.14) AEs: Not reported

*Preliminary results presented, recruitment continues.

All participants had neutropenia and thrombocytopenia during lymphodepleting chemotherapy, and one patient died from E. coli bacteremia three days after transfer of T-cells during a period of neutropenia. In this study, no correlation was measured demonstrating relationship between percentage of anti-NY-ESO-1 CD4+ or CD8+ T-cells at one month post transfer and disease response.

With evidence of activity for genetically engineered T-cells targeting NY-ESO-1, additional studies of genetically engineered T-cell receptors against NY-ESO-1 have been conducted. A Phase I, open-label trial of NY-ESO-1^c259^ T-cells (letetresgene autoleucel [lete-cel]; GSK3377794) included 45 patients with recurrent or metastatic SYN (NCT01343043) (29). This study resulted in one complete response (34 weeks) and 14 partial responses (29, 32). In this trial, four cohorts were established with varying NY-ESO-1 expression and lymphodepleting chemotherapeutic regimens. Cohort 1 included patients with high NY-ESO-1 expression and a high lymphodepletion regimen (fludarabine and cyclophosphamide). Cohort 2 included patients with low NY-ESO-1 expression with a high lymphodepletion regimen. Cohort 3 included patients with high NY-ESO-1 expression with a differing high lymphodepletion regimen (cyclophosphamide only). Cohort 4 included patients with high NY-ESO-1 expression and a low lymphodepletion regimen (dose reduced cyclophosphamide and fludarabine).

Cohorts 1-3 have complete data available as of January 2020. In Cohort 1, six of 12 patients had at least a partial response, one patient had a complete response, and the median overall survival (OS) was 24.3 months (29). In Cohort 2, four patients of 13 had partial response and the median OS was 9.9 months. In Cohort 3, one patient of 5 had a partial response with an OS of 19.9 months. In Cohort 1 all six responders had presence of anti-NY-ESO-1 T-cells at 6 months post cells transfer (39). More than 40% of patients in all cohorts had Grade 3 or higher hematologic Adverse Events (AEs) and 44% of patients had cytokine release syndrome, of which four were Grade 3 or higher (29). This is similar to toxicity seen for chimeric antigen receptor (CAR) T-cell therapy, where 69% of patients had Grade 3 or higher neutropenia and 92% of patients had cytokine release syndrome, of which 6% were Grade 3 or higher (40). A Phase II master protocol is currently in recruitment to test NY-ESO-1 T-cells for patients with metastatic SYN or myxoid/round cell liposarcoma who have progressed after standard treatment (NCT03967223) (**Table 2**) (41).

**Table 2:** Clinical trials in recruitment for treatment of SYN.

Target	Treatment	Population	Trial	Phase
NY-ESO-1	Autologous NY-ESO-1^c259^ T-cells	HLA- A*02, Previously untreated advanced SYN or myxoid/round cell liposarcoma	NCT03967223 (41)	II
	Anti-NY-ESO-1 T-cells with co-expression of CD8α chain Anti-NY-ESO-1 T-cells with co-expression of TGF-β Epigenetically reprogrammed NY-ESO-1 T-cells	HLA- A*02, Previously treated advanced SYN or myxoid/round cell liposarcoma	NCT04526509 (42)	I
	NY-ESO-1 aAVCs	Relapsed, refractory advanced solid tumors known to express NY-ESO-1	NCT04939701 (43)	I, II
PRAME	Anti-PRAME T-cells	HLA-A*02, Relapsed, refractory PRAME positive	NCT04262466 (44)	I, II
BRD9	BRD9 inhibitor (CFT8634)	Locally advanced or metastatic SMARCB1-perturbed cancers, including SYN	NCT05355753 (45)	I
	BRD9 inhibitor (FHD-609)	Advanced SYN or advanced SMARCB1-loss tumors	NCT04965753 (46)	I

Next generation NY-ESO-1 T-cell products may provide additional benefits, but qualification is needed. Currently, a Phase II master protocol of three different next generation NY-ESO-1 T-cell products is in recruitment for treatment of solid tumors with NY-ESO-1 expression (NCT04526509) (42).

CD8 is a cell surface glycoprotein that acts as a co-receptor with T-cell receptors and assists in T-cell binding to MHC1 (47, 48). Previous *in vitro* study has found that engineered T-cells targeting a cancer testis antigen that co-expressed CD8α led to greater CD4+ T-cell activity (49). One arm of the master protocol will use anti-NY-ESO-1 T-cells which co-express the CD8α chain to determine efficacy of this technology (GSK3901961) (42).

An additional technology of interest combines anti-NY-ESO-1 T-cells with a dominant negative transforming growth factor- β (TGF-β) type II receptor (GSK3845097) (42). TGF-β is a regulator of immune homeostasis and has been found to inhibit tumor cellular immunity (50, 51). T-cells genetically engineered to target prostate cancer combined with a dominant negative TGF-β receptor have been found to cause tumor regression and enhanced survival in a murine model (51). This technology may improve the tumor microenvironment by limiting the impact of immune down-regulators, specifically TGF- β, in treatment of SYN.

T-cell quality impacts success in ACT. Previous study of ex-vivo ACTs has found that stem-like surface markers on T-cells are more likely to lead to response and stem-like T-cells are more capable of *in vivo* expansion (52). This knowledge has led to the development of technology that improves the stem-like quality of engineered T-cell receptors ex vivo through epigenetic reprogramming (53). The third arm of the master protocol will assess anti-NY-ESO-1 T-cells after a proprietary epigenetic reprogramming process to enhance the stem-like quality of the T-cells (GSK4427296). Combining engineered T-cells with additional genetic modifications may enhance efficacy of ACT targeting NY-ESO-1. Beyond NY-ESO-1 targeted therapies, other ACTs against cancer testis antigens have been developed for the treatment of SYN.

### 2.2 PRAME adopted cell transfer

Preferentially expressed antigen in melanoma (PRAME) is a cancer testis antigen that is expressed in 95% of metastatic melanoma (54). It is also expressed homogenously in SYN at high levels (55). PRAME functions through inhibition of apoptosis and signal transduction of the retinoic acid receptor, causing tumorigenesis (56). Based on its expression and impact on sarcomagenesis, it is an additional target for directed engineered T-cell therapy.

The IMA203 trial utilized T- cell receptor engineered T-cells against PRAME in HLA-A*02:01 (NCT03686124) (57). This Phase I trial of 12 evaluable patients resulted in six patients with stable disease and six patients with partial response, three of whom had SYN (33). The most common adverse events were cytopenias, neurotoxicity, and cytokine release syndrome. One patient had a dose limiting toxicity. Another, currently recruiting, trial for treatment of advanced solid tumors with PRAME and HLA-A*02:01 expression will test IMC-F106C, a T-cell receptor against PRAME, both in combination with checkpoint inhibitors and as a single agent (NCT04262466) (44). Results of this trial are expected in 2024.

### 2.3 MAGE adopted cell transfer

Melanoma-associated antigen (MAGE) proteins are clustered on the X chromosome. Expression of MAGE protein is generally restricted to reproductive tissues. This protein functions by inhibition of p53 and thereby limits tumor suppression (58, 59). MAGE-A4 is a cancer testis antigen that is expressed in many tumor types including lung cancer (19-35%), breast cancer (13%), ovarian cancer (47%), colon cancer (22%), esophageal cancer (60%), and soft tissue sarcomas, including 50-80% of SYN (24, 60–62).

Afamitresgene autoleucel are autologous T-cells which are isolated from patients, transduced with a lentiviral vector containing the MAGE-A4^c1032^ T-cell receptor, and expanded prior to infusion. Recently, results of a Phase I dose-escalation and expansion trial of Afamitresgene autoleucel was conducted in patients who were HLA-A*02 positive with advanced cancers that expressed MAGE-A4 (NCT03132922). In this study, patients received lymphodepletion regimen of cyclophosphamide and fludarabine prior to Afamitresgene autoleucel infusion (34, 35).

In the Cohort 3/expansion group (28 patients), 7 of 28 patients had a partial response, 11 of 28 had stable disease, while 10 of 28 either had progressive disease or were not evaluable (34). Results of this study showed no dose limiting toxicities and the most common Grade 3 or higher AE (>30%) were hematologic, including lymphopenia, leukopenia, neutropenia, anemia, and thrombocytopenia. Two patients had trial related deaths due to aplastic anemia and cerebral vascular accident. Notably, all responses to therapy occurred in patients with SYN, perhaps emphasizing the validity of targeting MAGE-A4 in this histology.

A Phase II, single arm, open-label clinical trial of Afamitresgene autoleucel in patients with advanced SYN or myxoid/round cell liposarcoma (MRCLS) called SPEARHEAD-1 is currently underway (NCT04044768) (36). Preliminary results from SPEARHEAD-1 were presented at the 2022 American Society of Clinical Oncology Annual Meeting (37). Patients received Afamitresgene autoleucel and were evaluable for response (Phase I, n = 18; Phase II, n = 51) with all patients expressing the HLA-A*02 allele. The pooled investigator-assessed overall response rate was 36.2% which occurred across MAGE-A4 H-scores of 134-400. The median duration of response was 52 weeks (8.29 – 75.14). Response rate was higher in patients with fewer lines of previous therapy, smaller target lesions, higher MAGE-A4 scores, those without bridging therapy, women, patients over 40, and patients from North America. The SPEARHEAD-1 trial is currently recruiting for Cohort 2 which will specifically evaluate patients with SYN (36).

### 2.4 Challenges of cancer testis antigen ACT

There has been success in treating SYN through targeting NY-ESO-1, PRAME, and MAGE-A4 using ACT, with more trial results forthcoming. While this is laudable, there are challenges to the treatment of SYN using these technologies. One barrier is the restriction of many of these therapies to patients with HLA-A*02. Studies have found that HLA-A*02 is more common in Caucasian populations compared to African-American and Asian populations (63). Other barriers for ACT include the multi-week time needed for the production of genetically engineered T-cells, the pre-treatment lymphodepletion regimen which often requires hospitalization, and the high cost of therapy (64, 65). While many of these issues may be overcome through improvement in manufacturing techniques and health systems changes, some may be incontrovertible.

## 3 NY-ESO-1 artificial adjuvant vector cells

**Figure 2:**
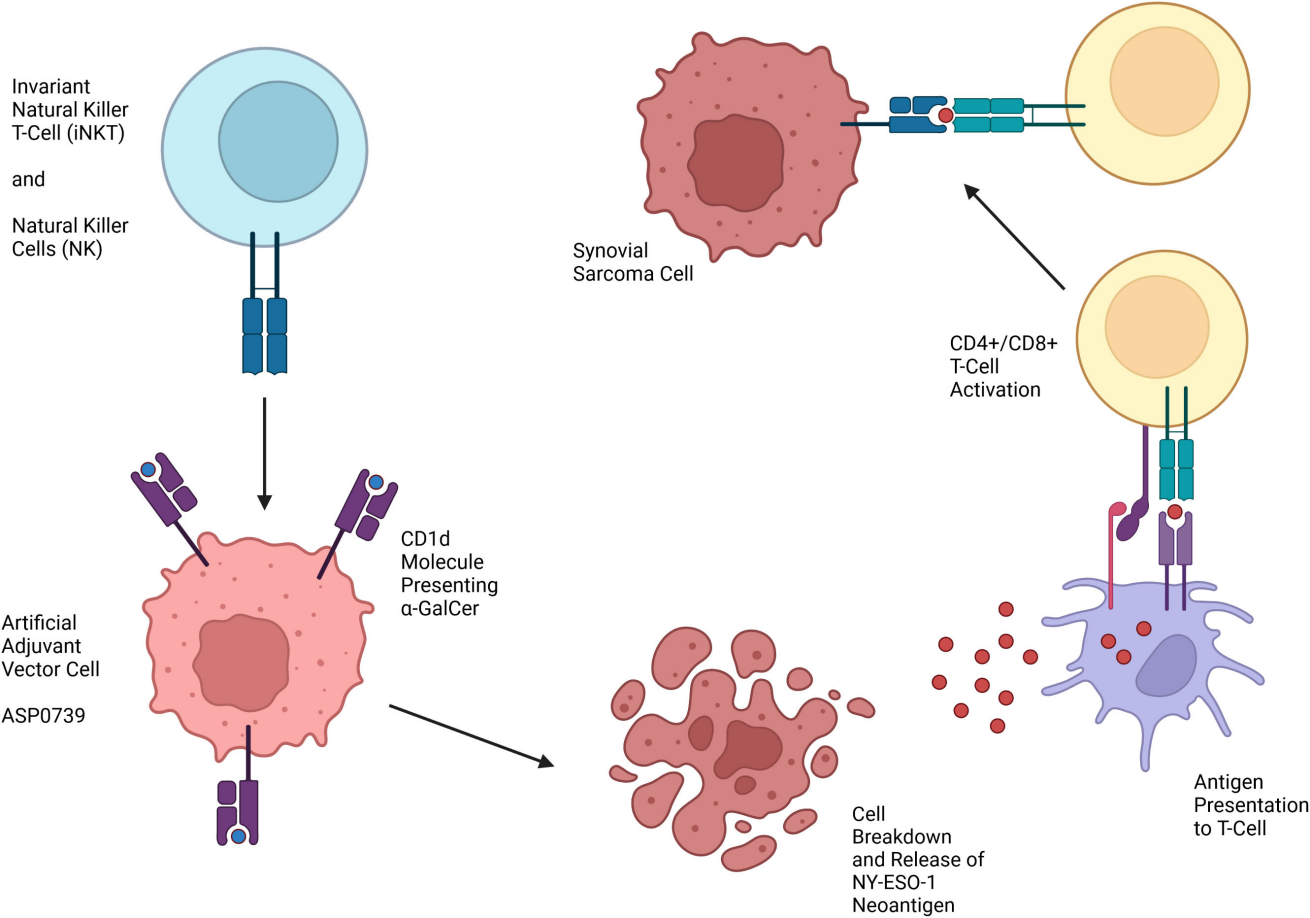
Mechanism of action of Artificial Adjuvant Vector Cells targeting NY-ESO-1 for treatment of SYN.

One technology in development for the treatment of SYN that does not require HLA matching is artificial adjuvant vector cells (aAVCs). aAVCs are loaded with an exogenous glycolipid ligand, α-galactosylceramide (α-GalCer), which is presented on a CD1d molecule and activates invariant natural killer T (iNKT) cells (**Figure 2**) (66). aAVCs also express a specific tumor-associated antigen. The α-GalCer synthetic ligand activating iNKT allows iNKT and natural killer (NK) cells to kill aAVCs, leading to the release of the tumor-associated antigen. Endogenous dendritic cells then serve as antigen presenting cells which allow for creation of CD4+ and CD8+ anti-tumor antigen T-cells. Previously, a Phase II trial of patients with non-small cell lung cancer infused with α-GalCer-pulsed Antigen Presenting Cells (APCs) showed efficacy (67). aAVCs that express NY-ESO-1 have been shown in a murine model to elicit NY-ESO-1 specific CD8+ T-cells as well as have an anti-tumor effect (68).

ASP0739 is an aAVC product targeting NY-ESO-1 being developed for treatment of SYN. Currently, a Phase I trial is in recruitment to test ASP0739 in patients with solid tumors including SYN, myxoid/round cell liposarcoma, ovarian carcinoma, non-small cell lung cancer, and esophageal squamous cell carcinoma (NCT04939701) (43). Phase II of the trial will use ASP0739 in combination with pembrolizumab, an antibody against PD-1 on lymphocytes that prevents de-activation of T-cells by tumors. While the results of these studies are yet to come, these trials will hopefully provide an additional therapeutic opportunity for treatment of SYN without the need for HLA matching.

## 4 BRD9 targeted therapy

**Figure 3:**
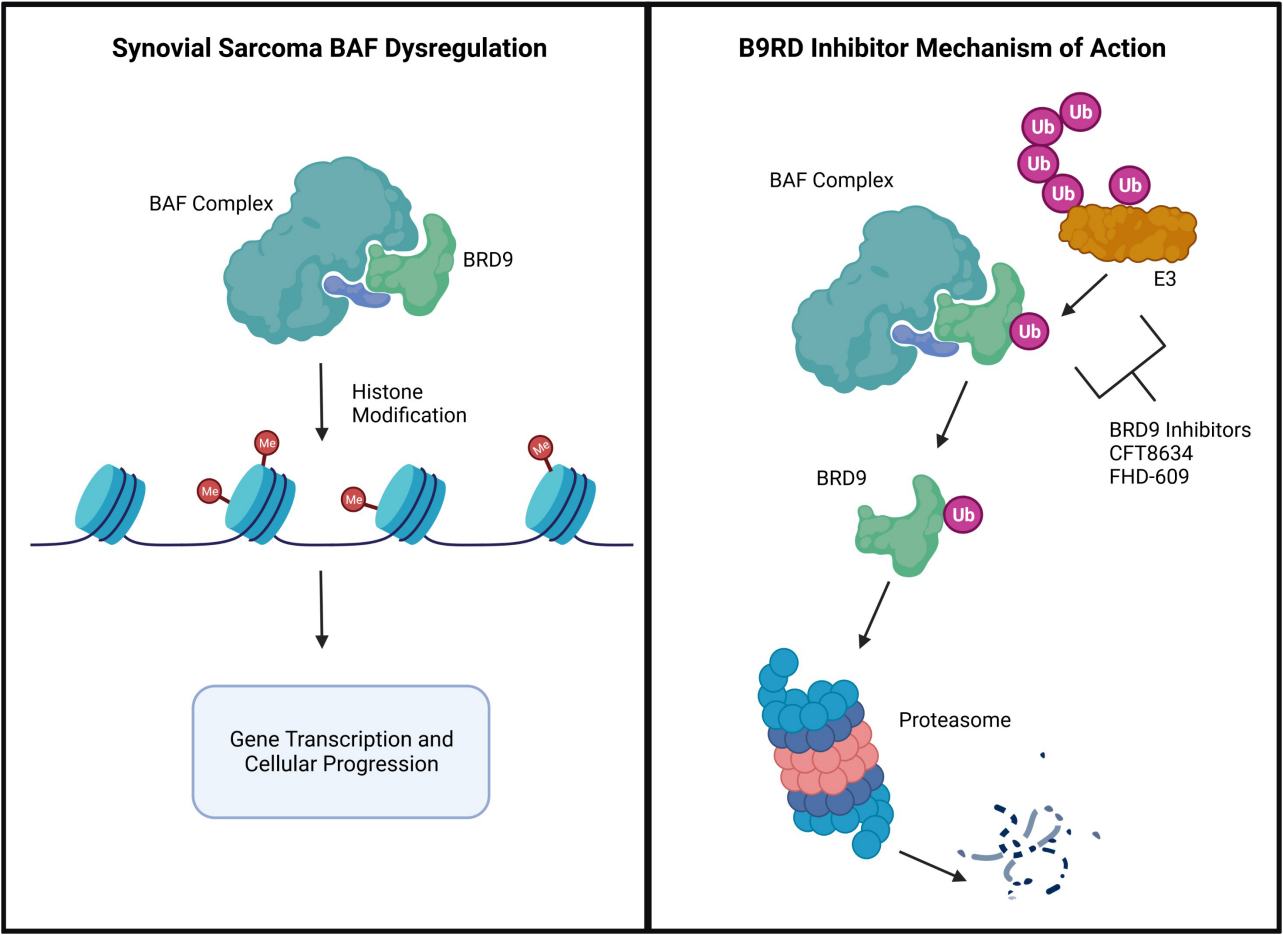
BRD9 inhibitor therapy for the treatment of SYN.

BRD9 small molecule inhibitors are currently in development for the treatment of SYN (**Figure 3**). Mammalian SWI/SNF (mSWI/SNF or BAF) complexes are chromatin remodelers that allow for alterations in gene expression and DNA transcription. SS18-SSX fusion oncoprotein has been found to hijack the BAF complex, displacing wild-type SS18, resulting in changes in transcription and thus the development of SYN (69). These findings have led to the recognition of BAF complexes and specific subunits as potential targets for treatment of SYN (70).

BRD9 is a non-BET bromodomain protein and subunit of BAF complexes that has been recognized as a potential target for cancer treatment. In 2017, the first BRD9 chemical degrader was created that bridges the BRD9 bromodomain and E3 ubiquitin ligase complexes *in vitro* (71). Since then, numerous BRD9 inhibitors and have been developed (72–75). Degradation of BRD9 inhibits SYN tumor progression in a murine model (76). Therefore, BRD9 inhibition and/or degradation is a potential target for treatment of SYN.

CFT8634 is an oral heterobifunctional degrader that bridges BRD9 with E3 ligase, causing ubiquitination and proteasomal degradation of BRD9 (77). FHD-609 is an intravenous BRD9 degrader that bridges BRD9 with cereblon (CRBN) E3 ubiquitin ligase substrate that leads to proteasomal degradation (78). These therapies are currently undergoing Phase I trials for patients with advanced SYN (45, 46). The results of these trials are anticipated as potential therapies for treatment of SYN.

## 5 Conclusion

While standard of care treatment of advanced and metastatic SYN remains anthracycline based chemotherapy, there are numerous technologies in development for the treatment of advanced and metastatic SYN. These technologies stem from improved understanding of the tumor antigen expression and molecular mechanisms behind SYN. Engineered T-cell receptor therapies targeting CTAs has shown success in early-stage trials. Optimization of these engineered TCR treatments is currently being studied, with efforts to enhance T-cell antigen binding, alter the tumor microenvironment, and improve the quality of T-cells used for treatment. Alternative therapies without the need for HLA matching that are currently in recruitment for Phase I trials include aAVCs and BRD9 inhibitors.

Reviewing the new targeted and cellular therapies shows the tremendous progress that has been made over the preceding decades. Nonetheless, further study and qualification are required to ensure that we are doing the best for our patients. We anticipate that with the accelerated pace of discovery and application of new agents, treatment for patients with SYN will make remarkable strides in the upcoming years.

## Author contributions

MA conceptualized the manuscript. JRF and JWF wrote the original draft. JRF, JWF, BS, and MA were responsible for writing and editing subsequent drafts and providing final approval for the manuscript. All authors contributed to the article and approved the submitted version.
